# Supplementary material for: Resolving the full spectrum of human genome variation using Linked-Reads
Source: Genome Res. 2019 Apr;29(4):635–45. doi: 10.1101/gr.234443.118 (PMC6442396; doi:10.1101/gr.234443.118)
Supplement: Supplemental Material [file supp_gr.234443.118_Supplemental_longranger-2.2.zip › longranger-2.2.tar.bz2!/lr2.2-public/lib/stan/cmdstan/stan/doc/api/html/gaussian__dlm__obs_8hpp.html]

Stan: src/stan/prob/distributions/multivariate/continuous/gaussian\_dlm\_obs.hpp File Reference


|  |  |
| --- | --- |
|  | Stan  2.5.0  probability, sampling & optimization |


- Main Page
- Related Pages
- Namespaces
- Classes
- Files

- File List
- File Members

All Classes Namespaces Files Functions Variables Typedefs Enumerations Enumerator Friends Macros Pages

- src
- stan
- prob
- distributions
- multivariate
- continuous

Namespaces |
Functions

gaussian\_dlm\_obs.hpp File Reference

`#include <boost/random/normal_distribution.hpp>`  
`#include <boost/random/variate_generator.hpp>`  
`#include <stan/math/matrix_error_handling.hpp>`  
`#include <stan/math/error_handling.hpp>`  
`#include <stan/math/error_handling/dom_err.hpp>`  
`#include <stan/prob/constants.hpp>`  
`#include <stan/prob/traits.hpp>`  
`#include <stan/agrad/rev.hpp>`  
`#include <stan/meta/traits.hpp>`  
`#include <stan/agrad/rev/matrix.hpp>`  
`#include <stan/agrad/rev/matrix/to_var.hpp>`  
`#include <stan/math/matrix/add.hpp>`  
`#include <stan/math/matrix/dot_product.hpp>`  
`#include <stan/math/matrix/inverse_spd.hpp>`  
`#include <stan/math/matrix/log.hpp>`  
`#include <stan/math/matrix/log_determinant_spd.hpp>`  
`#include <stan/math/matrix/multiply.hpp>`  
`#include <stan/math/matrix/quad_form.hpp>`  
`#include <stan/math/matrix/subtract.hpp>`  
`#include <stan/math/matrix/tcrossprod.hpp>`  
`#include <stan/math/matrix/trace_quad_form.hpp>`  
`#include <stan/math/matrix/transpose.hpp>`

Go to the source code of this file.

|  |  |
| --- | --- |
| Namespaces | |
|  | stan |
|  | Probability, optimization and sampling library. |
|  | |
|  | stan::prob |
|  | Templated probability distributions. |
|  | |

|  |  |
| --- | --- |
| Functions | |
| template<bool propto, typename T\_y , typename T\_F , typename T\_G , typename T\_V , typename T\_W , typename T\_m0 , typename T\_C0 > | |
| return\_type< T\_y, typename   return\_type< T\_F, T\_G, T\_V,   T\_W, T\_m0, T\_C0 >::type >  ::type | stan::prob::gaussian\_dlm\_obs\_log (const Eigen::Matrix< T\_y, Eigen::Dynamic, Eigen::Dynamic > &y, const Eigen::Matrix< T\_F, Eigen::Dynamic, Eigen::Dynamic > &F, const Eigen::Matrix< T\_G, Eigen::Dynamic, Eigen::Dynamic > &G, const Eigen::Matrix< T\_V, Eigen::Dynamic, Eigen::Dynamic > &V, const Eigen::Matrix< T\_W, Eigen::Dynamic, Eigen::Dynamic > &W, const Eigen::Matrix< T\_m0, Eigen::Dynamic, 1 > &m0, const Eigen::Matrix< T\_C0, Eigen::Dynamic, Eigen::Dynamic > &C0) |
|  | The log of a Gaussian dynamic linear model (GDLM). More... |
|  | |
| template<typename T\_y , typename T\_F , typename T\_G , typename T\_V , typename T\_W , typename T\_m0 , typename T\_C0 > | |
| return\_type< T\_y, typename   return\_type< T\_F, T\_G, T\_V,   T\_W, T\_m0, T\_C0 >::type >  ::type | stan::prob::gaussian\_dlm\_obs\_log (const Eigen::Matrix< T\_y, Eigen::Dynamic, Eigen::Dynamic > &y, const Eigen::Matrix< T\_F, Eigen::Dynamic, Eigen::Dynamic > &F, const Eigen::Matrix< T\_G, Eigen::Dynamic, Eigen::Dynamic > &G, const Eigen::Matrix< T\_V, Eigen::Dynamic, Eigen::Dynamic > &V, const Eigen::Matrix< T\_W, Eigen::Dynamic, Eigen::Dynamic > &W, const Eigen::Matrix< T\_m0, Eigen::Dynamic, 1 > &m0, const Eigen::Matrix< T\_C0, Eigen::Dynamic, Eigen::Dynamic > &C0) |
|  | |
| template<bool propto, typename T\_y , typename T\_F , typename T\_G , typename T\_V , typename T\_W , typename T\_m0 , typename T\_C0 > | |
| return\_type< T\_y, typename   return\_type< T\_F, T\_G, T\_V,   T\_W, T\_m0, T\_C0 >::type >  ::type | stan::prob::gaussian\_dlm\_obs\_log (const Eigen::Matrix< T\_y, Eigen::Dynamic, Eigen::Dynamic > &y, const Eigen::Matrix< T\_F, Eigen::Dynamic, Eigen::Dynamic > &F, const Eigen::Matrix< T\_G, Eigen::Dynamic, Eigen::Dynamic > &G, const Eigen::Matrix< T\_V, Eigen::Dynamic, 1 > &V, const Eigen::Matrix< T\_W, Eigen::Dynamic, Eigen::Dynamic > &W, const Eigen::Matrix< T\_m0, Eigen::Dynamic, 1 > &m0, const Eigen::Matrix< T\_C0, Eigen::Dynamic, Eigen::Dynamic > &C0) |
|  | The log of a Gaussian dynamic linear model (GDLM) with uncorrelated observation disturbances. More... |
|  | |
| template<typename T\_y , typename T\_F , typename T\_G , typename T\_V , typename T\_W , typename T\_m0 , typename T\_C0 > | |
| return\_type< T\_y, typename   return\_type< T\_F, T\_G, T\_V,   T\_W, T\_m0, T\_C0 >::type >  ::type | stan::prob::gaussian\_dlm\_obs\_log (const Eigen::Matrix< T\_y, Eigen::Dynamic, Eigen::Dynamic > &y, const Eigen::Matrix< T\_F, Eigen::Dynamic, Eigen::Dynamic > &F, const Eigen::Matrix< T\_G, Eigen::Dynamic, Eigen::Dynamic > &G, const Eigen::Matrix< T\_V, Eigen::Dynamic, 1 > &V, const Eigen::Matrix< T\_W, Eigen::Dynamic, Eigen::Dynamic > &W, const Eigen::Matrix< T\_m0, Eigen::Dynamic, 1 > &m0, const Eigen::Matrix< T\_C0, Eigen::Dynamic, Eigen::Dynamic > &C0) |
|  | |


---

[ Stan Home Page ]

*© 2011–2014,
Stan Development Team.*
